# Molecular entanglement can strongly increase basicity

**DOI:** 10.1038/s42004-024-01205-3

**Published:** 2024-05-28

**Authors:** Giorgio Capocasa, Federico Frateloreto, Matteo Valentini, Stefano Di Stefano

**Affiliations:** Department of Chemistry Università di Roma La Sapienza and ISB-CNR Sede Secondaria di Roma - Meccanismi di Reazione P.le A. Moro 5, I-00185 Roma, Italy

**Keywords:** Interlocked molecules, Self-assembly

## Abstract

Brønsted basicity is a fundamental chemical property featured by several kinds of inorganic and organic compounds. In this Review, we treat a particularly high basicity resulting from the mechanical entanglement involving two or more molecular subunits in catenanes and rotaxanes. Such entanglement allows a number of basic sites to be in close proximity with each other, highly increasing the proton affinity in comparison with the corresponding, non-entangled counterparts up to obtain superbases, properly defined as *mechanically interlocked superbases*. In the following pages, the development of this kind of superbases will be described with a historical perusal, starting from the initial, serendipitous findings up to the most recent reports where the strong basic property of entangled molecular units is the object of a rational design.

## Abstract (web summary)

Brønsted basicity can be greatly enhanced by the mechanical entanglement of two or more interlocked molecular subunits within catenanes and rotaxanes. Here, the authors discuss the development of such mechanically interlocked superbases, and outline challenges and opportunities for future directions of research.

## Introduction

More than 60 years after Wassermann first obtained evidence for the formation of an artificial mechanically interlocked molecule (MIM)^1–3^, entangled systems still captivate a great interest in different disciplines by virtue of their fascinating architectures, as well as the nontrivial topologies conferred to some MIMs by the mechanical bond^1,4,5^.

The mechanical bond is ubiquitous in biological systems, where it imparts function to DNA^6–8^ and proteins^9^ and is key to building some of the artificial molecular machines that earned the 2016 Nobel Prize to Sauvage^10^, Feringa^11^, and Stoddart^12^. An interlocked compound often displays properties that are not shared with an unentangled molecule. For example, mechanically entangled polymers display markedly different rheological properties compared to their non-interlocked counterparts^13–15^, and molecular knot complexes with transition metals can have far more elaborated second coordination spheres than their unentangled analogs^16^. Brønsted basicity, one of the main chemical properties that can be tuned by molecular entanglement, is the focus of this present Review.

**Fig. 1:**
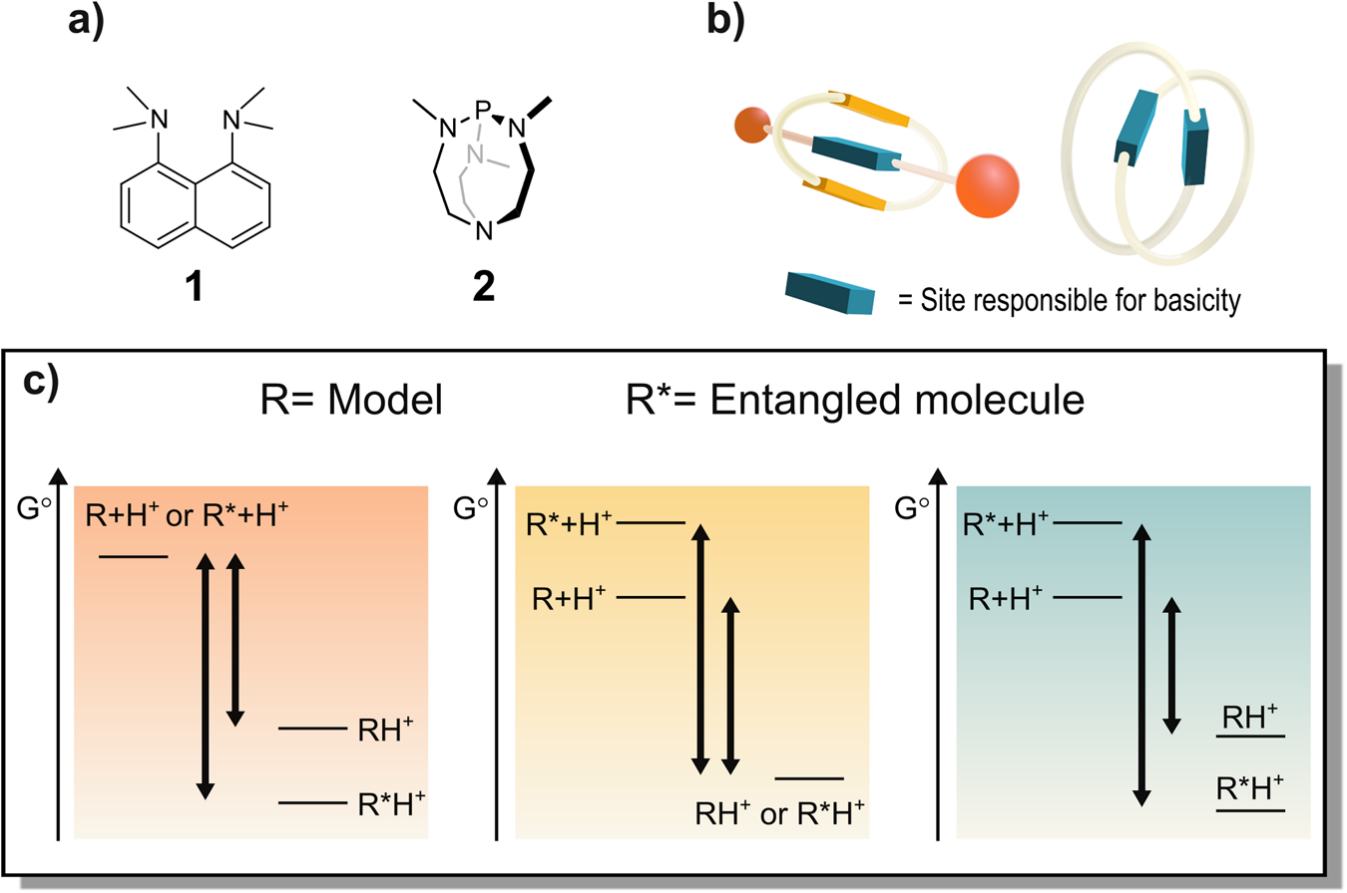
Basicity enhancement in classical superbases and in entangled molecules. **a** Structures of compounds that exhibit an increase in basicity due to the assistance of an auxiliary group which is part of the same network of covalent bonds as the basic site. **b** A rotaxane and a catenane which display an increase in basicity due to the mechanical bond between the subunit featuring the basic site and the one endowed with the auxiliary group. **c** Energy diagrams for the protonation of a generic base whose conjugate acid is stabilized by the molecular entanglement (left), whose basic form is destabilized by the molecular entanglement (typically observed case, center), and where the two effects concur in increasing the basicity (right). To a given difference in ∆G° corresponds a specific ∆pK_a_H.

Basicity has long been known to be influenced by the co-presence of multiple basic sites located in near proximity. Such base moieties (two or more) can belong to the same network of covalent bonds or be part of two subunits held together by a mechanical bond. As such, 1,8-bis(dimethylamino)naphthalene **1** and its derivatives^17^, as well as proazaphosphatranes such as Verkade’s superbase **2** display an increase in basicity due to the assistance of auxiliary groups located on the same backbone as the basic site (see Fig. 1a)^18^. This property is not unique to artificial molecules. Biological systems often achieve changes in the pK_a_ (or pK_a_H, which indicates the pK_a_ of the conjugated acid of a given base) of ionizable groups that are part of proteins or nucleic acids. Amongst the factors that give rise to this effect is the presence of auxiliary groups in close spatial proximity to the ionizable site. The resulting pK_a_ (or pK_a_H) changes can be major (ΔpK_a_ > 2) and are key to the catalytic activity of enzymes at physiological pH^19^. On the other hand, proximity through a mechanical bond among the base functionalities also produces an increase in basicity (see Fig. 1b). Since a mechanical bond is not limited by valence rules, its understanding paves the way to the designing of new classes of bases, where a proton is hosted by one or more neighboring groups through novel molecular architectures. A mechanical bond is not limited by valence rules in the sense that there is no fixed ratio between the numbers of mechanically interconnected units in a MIM. Therefore, one cannot draw valence rules such as “an uncharged, coordinatively saturated nitrogen atom preferentially makes three bonds” as is for the case of the covalent chemical bond. Furthermore, unless there is an attractive interaction between the components of a mechanically interlocked molecule, the mechanical bond angles and lengths are poorly defined in solution due to the co-conformational freedom exhibited by the subunits that comprise the MIM.

Bases that are stronger than **1** in acetonitrile are often referred to as *superbases*^20^. Organic superbases have complementary properties to strong inorganic bases that are desirable for organic synthesis such as a well-defined stoichiometry and usually low nucleophilicity. Furthermore, due to their neutral charge, organic superbases are usually soluble in the most common organic solvents while retaining their high basicity. In contrast, ionic inorganic bases tend to form ion pairs and aggregates with reduced protophilicity^21^. Amongst the drawbacks of organic superbases, we cite their high molecular weights and their synthesis that are often arduous compared to inorganic bases, leading to low atom economy and sustainability issues. Nevertheless, the use in catalytic amounts bypasses these issues and allows the application of such reagents to organic synthesis^22^, asymmetric catalysis^23–25^, or as ligands for transition metals^26–28^. Additionally, superbases can be employed in environmental remediation strategies such as CO_2_ splitting^29^ and capture^30–32^.

This Review showcases a number of mechanically interlocked molecules whose basicity is highly increased with respect to the corresponding non-entangled molecules with the same basic site. The synthesis of such compounds and the discussion on the increase in basicity are reported. Since gas basicity data is unavailable for most of the species here treated and pK_a_ or pK_a_H values depend on the solvent system used, the molecules are not reported in order of basicity. Indeed, gas basicity is a measure of the intrinsic basicity of a given molecule while pK_a_H values reflect the relative stabilities of the base and its conjugate acid in the solvent system used for the measurement^33^. Instead, the increase in basicity due to the molecular entanglement is expressed as the difference in pK_a_H of the interlocked molecule *versus* that of an appropriate, unentangled model in the same solvent.

All increases in basicity are due to a relative stabilization of the protonated state of the molecule with respect to its non-protonated state (see Fig. 1c), which translates into an increase in pK_a_H. This can be thought of as a stabilization of the protonated state (e.g.: *via* hydrogen bonding or sharing of the proton between more basic sites) with respect to the same protonated state that does not enjoy such stabilization or as a destabilization of the non-protonated state (e.g.,: *via* the establishment of unfavorable Van der Waals interactions between parts of the molecule or solvent exclusion from a cavity) compared to a model (see Fig. 1c). Furthermore, both a stabilization of the protonated state and a destabilization of the non-protonated state may occur at the same time.

All evidence collected so far around the enhancement in basicity for interlocked systems points towards a destabilization of the unprotonated state, as is generally observed in the case of increased binding when dealing with preorganized structures^34^. However, a contribution from the stabilization of the protonated species cannot be excluded a priori.

Due to the huge number and variety of *catenane* and *rotaxane* structures, it is not surprising that most of the time enhancements of basicity due to molecular entanglement were serendipitously found in this class of molecules.

### Entanglement-driven enhancement of basicity in catenanes

Catenanes are constituted of two or more macrocycles, interlocked in a manner resembling the links of a chain. In principle, there are no limitations on the functional groups that can be included in the macrocycle units. Indeed, catenane rings can be organic or inorganic in nature^35^. As such, several examples of catenanes featuring basic sites are published in the literature. However, data on the pK_a_H variations of such groups compared to those present in an unentangled molecule is scarce.

**Fig. 2:**
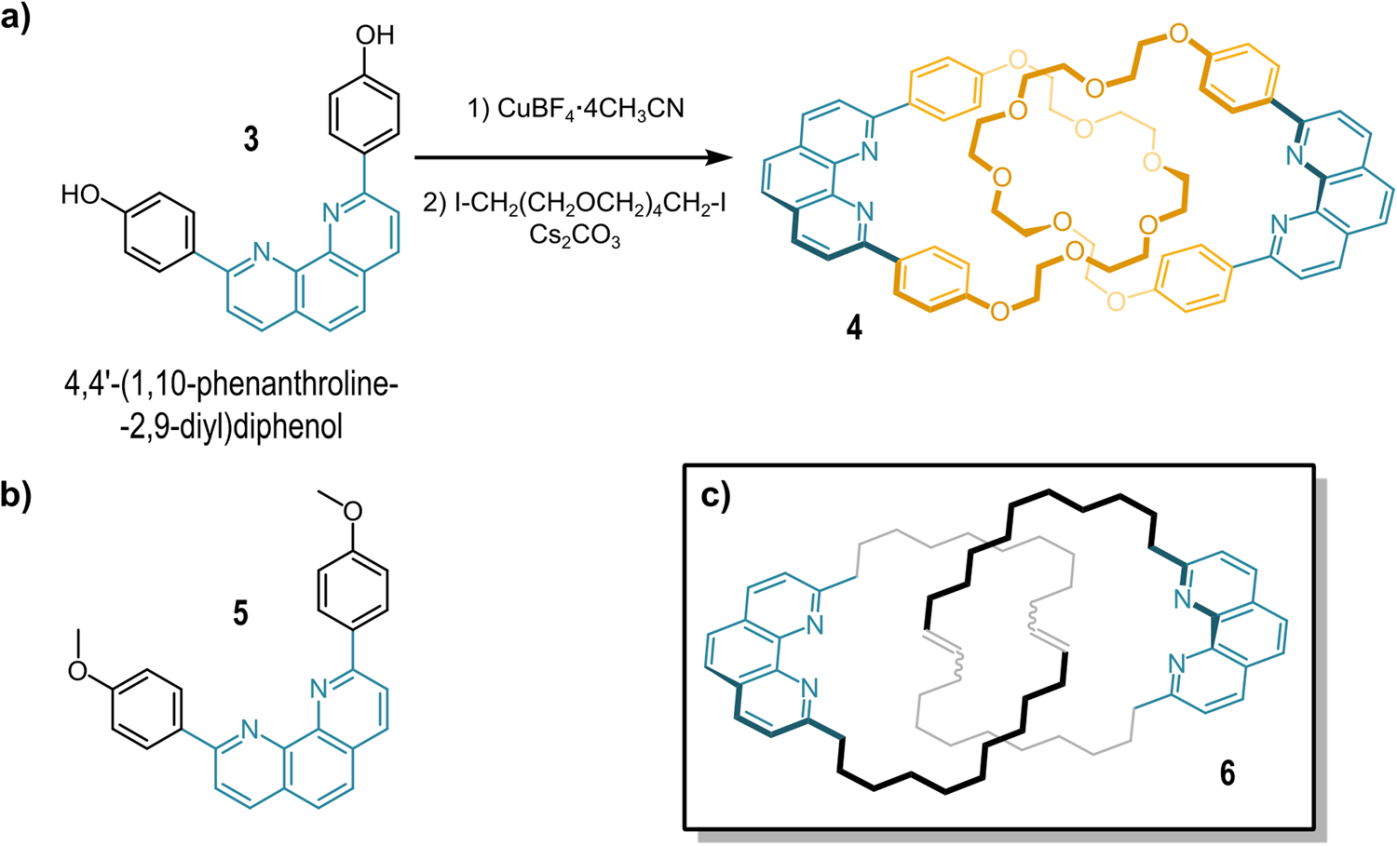
Catenand 4 and its open-chain analogue. **a** Templated synthesis of catenand **4**. **b** Structure of **5**, the open-chain model of catenand **4**. **c** Structure of catenand **6** obtained by Grubbs metathesis.

In 1983, the Sauvage group reported the first practical, templated synthesis of [2]*catenand* **4** constituted of two identical 1,10-phenanthroline-based macrocycles^36–38^. The scheme is reported in Fig. 2a. A *catenand* is the catenane ligand devoid of the metal ion while a *catenate* is a metalated catenand. The synthesis employs a copper(I) salt to preorganize the two phenanthroline units bearing 4-phenol moieties in a tetrahedral arrangement. Then, the macrocycles are closed one around the other through a Williamson etherification with pentaethylene glycol diiodide (yield 27%). The resulting copper (I) catenate could be demetalated to obtain the free catenand **4** in 19% overall yield from the 1,10-phenanthroline starting material. Later, exploiting a ring-closing olefin metathesis reaction on a slightly modified intermediate bearing terminal double bonds, the group was able to improve the yield of the catenate-forming reaction up to 92%. Reduction of the double bond and demetallation of the catenate afforded **4** in 51% yield from 1,10-phenanthroline^38^.

Not being constricted to a particular geometry, the mechanical bond imparts the ligand enough flexibility to efficiently coordinate several cations in a 1:1 molar ratio despite their difference in ionic radii. Stable metal catenates of **4** with several metal ions such as Cu(II), Ag(I), Co(II), Zn(II), Cd(II), Li(I)^39^, and Ni(I)^40^ were subsequently synthesized and characterized.

Upon binding of the metal to yield the metal catenate, the ligand backbone undergoes a conformational rearrangement compared to the free catenand^41^. While in **4** the phenanthroline moieties are expected to be far apart, in the metal complex they both participate in chelating the ion. Moreover, the coordination of the metal cation makes each phenanthroline unit even more electron-poor, so that they engage in stabilizing π-π interactions with the phenoxylene unit on the other macrocycle^41^. That is, the phenanthroline units located on the two interlocked macrocycles act together in the binding, stabilizing the complex through a chelate effect even though the nitrogen sites are not bound together by chains of covalent bonds.

Remarkably, in 1986 Sauvage et al. reported that **4** also strongly binds the proton^42^, the smallest existing cation.

As demonstrated by X-ray diffraction data, the proton and Cu(I) catenates share a similar structure, although the proton is bound to a single phenanthroline moiety, and not shared equally between the four nitrogen basic sites^42^. Regardless, the complexation of a single proton makes each of the two phenanthrolines sufficiently electron-poor to engage in favorable π-π interactions with the electron-rich phenoxylene rings, as seen for the Cu(I) catenate. In solution, the proton is rapidly exchanged between the nitrogen sites, as suggested by the highly symmetrical ^1^H-NMR spectrum of **4**H^+^ at room temperature. Conversely, there is no indication that the open-chain analog **5** (Fig. 2b) forms protonated dimers in acidic solutions.

The pK_a_H of **4**H^+^ was measured to be 8.5 through a competition method, to be compared to 5.1 of the topologically trivial model **5**H^+^. The authors employed a morpholine buffer in 70/30 CD_2_Cl_2_/CD_3_CN. Upon introduction of a small quantity of **4** or **4**H^+^ to this buffered solution, one can measure the [**4**H^+^]/[**4**] ratio and compute the pK_a_H of **4** by applying Eq. (1)

1 $${{pK}}_{a}{{{{{\rm{H}}}}}}\left({{{{{\bf{4}}}}}}\right)={{pK}}_{a}{{{{{\rm{H}}}}}}\left({morpholine}\right)+\log \frac{[{{{{{\bf{4}}}}}}{H}^{+}][{morpholine}]}{[{{{{{\bf{4}}}}}}][{morpholine}{H}^{+}]}$$

When the [morpholine]/[morpholineH^+^] ratio was set to 38/62, the ratio between [**4**H^+^] and [**4**] was measured to be 0.94 by NMR. The authors used a value of 8.7 for the pK_a_H of morpholine to obtain the reported pK_a_H(**4**) of 8.5.

This 2500-fold increase in basicity was attributed to the intramolecular stacking uniquely allowed by the mechanical bond in the catenate, and not present in the open-chain model. An additional indication of the importance of π-π stacking (and therefore of the molecular entanglement) in determining the structure of the proton catenate is given by the fact that **4**H^+^ can be further protonated to yield the diprotonated catenand **4**·2H^+^. ^1^H-NMR data shows that in **4**·2H^+^ the phenanthrolines still face each other despite both bearing a positive charge. The electrostatic repulsion of the two protonated basic sites is not enough to overcome the stabilizing effect of the stacking interactions, which are now enhanced by the protonation of both phenanthrolines.

Four decades later, Di Stefano and coworkers reported the Cu(I)-templated synthesis of a phenanthroline-based catenand (**6**) devoid of the phenoxylene moiety (Fig. 2c) through Grubbs metathesis^43–45^. As seen for **4**, such catenand strongly binds Cu(I) ions with the nitrogen atoms disposed in a tetrahedral fashion around the metal. Furthermore, the free catenand can also be protonated to yield a species whose ^1^H-NMR spectrum strongly resembles that of **6**·Cu^+^ ^46^. The authors reported that the proton catenate could not be deprotonated in any observable (^1^H-NMR) extent even by an excess of dibutylamine (50 mol equivs Bu_2_NH; pK_a_H = 11.25) in dichloromethane. However, unlike **4**, upon double protonation, **6** forms a species whose ^1^H-NMR has the same features as the free catenand. Since the two protonated phenanthroline moieties are not held together by stacking interactions, they repel each other in the di-cation state conferring high conformational mobility to the catenate.

### Entanglement-driven enhancement of basicity in rotaxanes

**Fig. 3:**
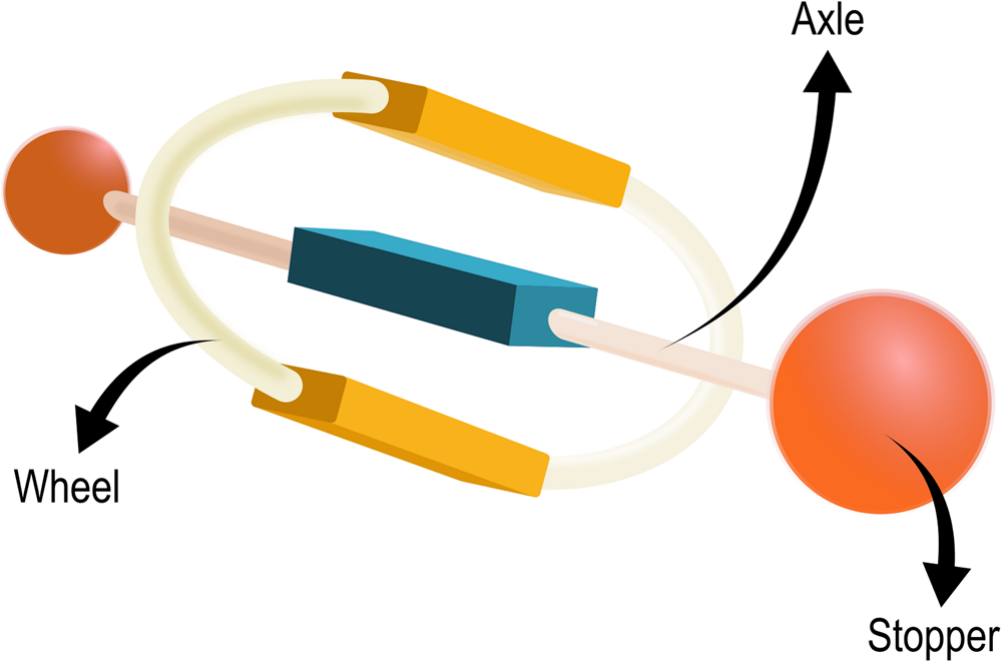
A rotaxane. Pictorial representation of a generical rotaxane.

The other prototypical class of mechanically interlocked molecules is represented by rotaxanes. Rotaxanes consist of two main molecular components: a rod-shaped molecule, the axle, threaded through a macrocycle, the wheel. The presence of two bulky groups – the stoppers - at the ends of the axle traps the wheel, preventing its release (see Fig. 3). A rotaxane made up of *n* subunits (counting all wheels and axles) is referred to as a [*n*]rotaxane.

Although rotaxanes display an enormous range of structural diversity, any enhancement of basicity due to molecular entanglement is rarely commented on by the authors. Indeed, it appears that basicity enhancements are often found serendipitously. Herein, we report basicity increases displayed by rotaxanes based on a bis-benzylamine moiety surrounded by a crown ether wheel. However, there is no reason to believe that such enhancements are peculiar only to this class of rotaxanes.

There exist a number of strategies exploiting supramolecular interactions to preorganize the components, and eventually afford rotaxanes in synthetically useful amounts. Examples of these interactions are coulombic interactions, metal coordination, hydrophobic forces, and hydrogen bonding^47–60^.

It is worth noting that the above-mentioned active templated synthesis of rotaxanes proceeds under kinetic control, often leading to the formation of a weakly bound pseudo-rotaxane (that is a rotaxane analog lacking one or both stoppers at the ends of the axle), where the wheel and the axle do not interact strongly. The increase in basicity in MIMs correlates with the formation of these weakly bound structures, whose protonation produces much more stable compounds, precisely due to the establishment of strong interactions between the entangled components^61–64^.

**Fig. 4:**
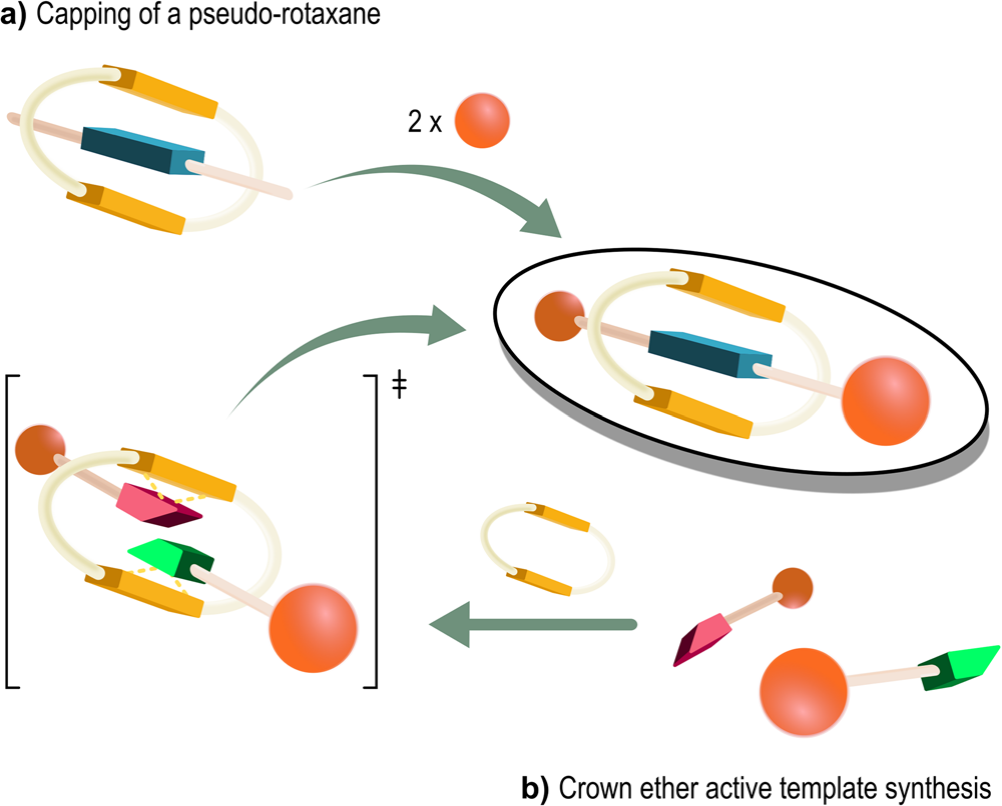
Hydrogen bonds in the synthesis of a rotaxane. Two synthetic strategies involving hydrogen bond interactions, for the formation of a rotaxane. **a** Capping of a pseudo-rotaxane. **b** Crown ether active template synthesis.

The synthetic strategies that involve hydrogen bonding, however, are related to the purpose of the present Review since such interaction at first facilitates the formation of the rotaxane, and is still present in the protonated rotaxane product. In a first strategy the hydrogen bond is used to pre-form a pseudo-rotaxane, whose axle is subsequently capped at the ends by large stoppers (Fig. 4a). In a second strategy, the crown ether catalyzes the alkylation of the benzylamine precursor, templating the formation of the rotaxane *via* hydrogen bond interactions (Fig. 4b). However, such strategies always yield the desired rotaxane as an ammonium salt with the crown ether interacting, through multiple hydrogen bonds, with the protonated nitrogen on the axle.

**Fig. 5:**
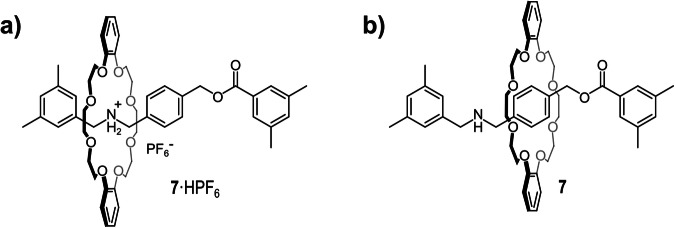
Rotaxane 7. Structures of **a** [2]rotaxane **7·**HPF_6_, and **b** its deprotonated form **7**.

The first examples of crown ether-based rotaxane with a secondary-ammonium moiety on the axle appeared in the literature in the second half of the nineties^65,66^. Concurrently, the researchers noticed some difficulties in obtaining the corresponding neutral species. Somehow, the neutralization of bis-benzylammonium, crown ether-based rotaxanes was not as straightforward as expected. In 1998, a successful deprotonation of an ammonium-based rotaxane was achieved by Balzani, Stoddart et al.^67^, but a second positively charged station present in the axle facilitated the process providing an alternative binding site for the crown ether. Two years later, Takata and coworkers studied more in detail the “*unusually lowered acidity*” of the secondary bis-benzylammonium group, present in the rotaxane **7·**HPF_6_ depicted in Fig. 5a, where the wheel is a dibenzo-24-crown-8 ether^68^. In such study, a great effort was devoted to the deprotonation of the ammonium moiety of the rotaxane to obtain its neutral counterpart. They found the neutralization of **7·**HPF_6_ practically impossible, even using harsh conditions. **7·**HPF_6_ was not deprotonated by treatment with potassium carbonate, triethylamine, diazabicycloundecene (DBU), or calcium hydride. Conversely, the corresponding pseudo-rotaxane **7**^**pseudo**^**·**HPF_6_, lacking the 3,5-dimethylbenzoyl stopper was rapidly neutralized by all of the above basic treatments, underlining the important role of the mechanical bond in the increased basicity of the secondary-ammonium group present along the axle. In the same work, it was also reported that such strongly increased basicity is a common feature of a series of previously synthesized rotaxanes having similar structures^69,70^. In addition, exchange experiments in the presence of deuterated water showed a very low kinetic acidity of **7·**HPF_6_. In contrast, the above-mentioned pseudo-rotaxane **7**^**pseudo**^**·**HPF_6_ rapidly exchanged the proton with the deuteron of D_2_O. Although the neutralization of **7·**HPF_6_ was not attained, the authors managed to “trap” the deprotonated form of **7** in the form of its *N*-acylated derivatives by treating **7·**HPF_6_ with various acylating agents in the presence of triethylamine as the base, in different solvents. In particular, they observed that, although the HPF_6_ salt of **7** is an ionic compound, it is more mobile on silica gel TLC than the neutral *N*-acetylated **7** (**7**^**Ac**^). This somehow suggested that **7·**HPF_6_ does not behave as a typical polar compound. The crystal structure of **7**^**Ac**^ showed that the crown ether wheel is located on the central aryl group, away from the *N*-acetylated moiety, and that no significant stabilizing interactions occur between the crown ether and the axle^71^.

**Fig. 6:**
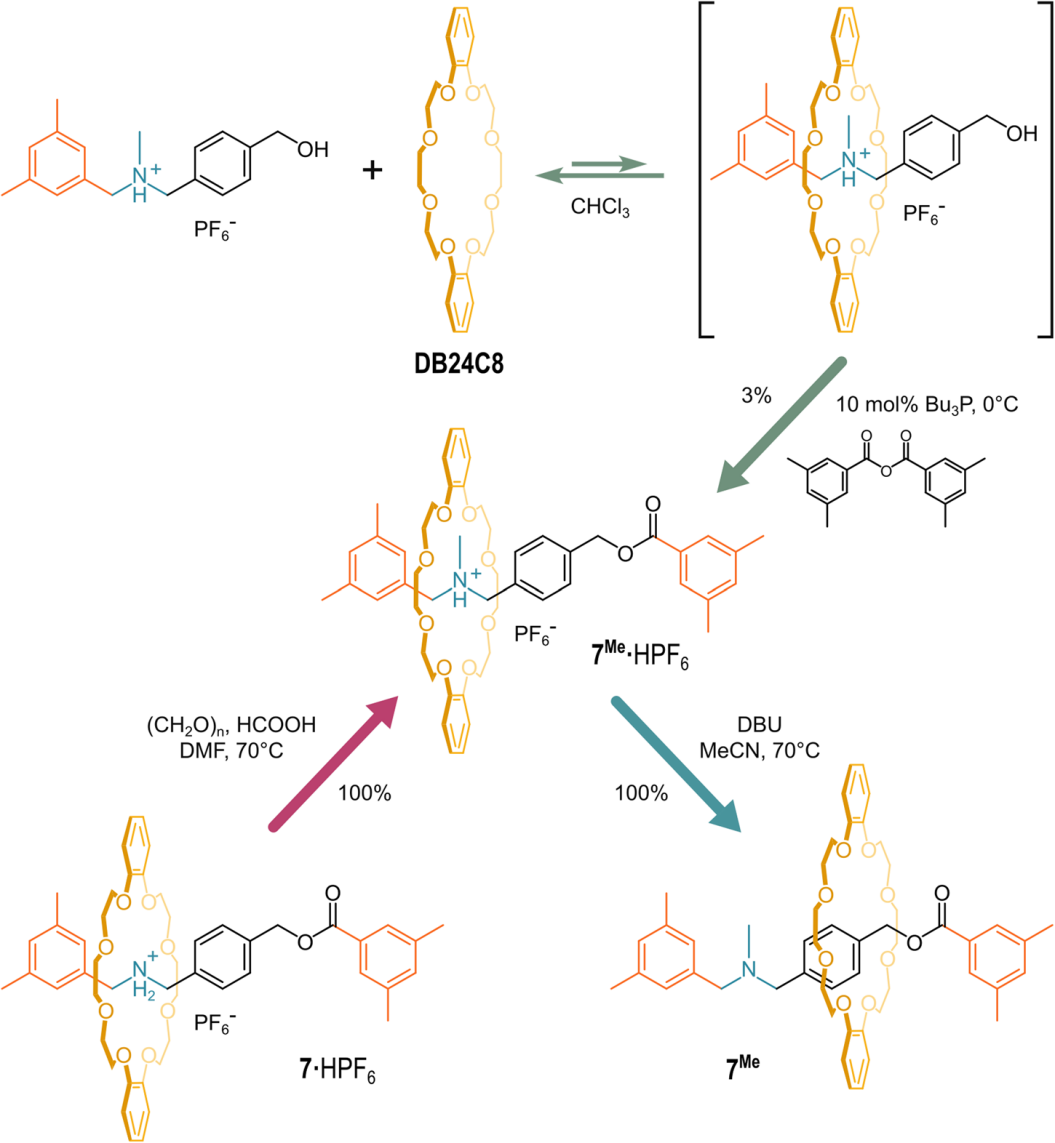
Deprotonation of a tertiary-amine-based rotaxane. Synthetic pathways to obtain **7**^**Me**^**·**HPF_6_ and its deprotonation with DBU.

In 2008, the same group successfully prepared the *N*-methylated derivative of **7** (**7**^**Me**^), (Fig. 6), either starting from the pseudo-rotaxane composed by the tertiary ammonium axle and the crown ether wheel (yield 3%) or performing a direct *N*-methylation of **7·**HPF_6_ *via* the Eschweiler–Clarke reaction (quantitative yield)^72^. The poor yield of the first reaction was likely due to the low affinity of the crown ether macrocycle for the tertiary ammonium axle, which is in accordance with the much lower basicity of **7**^**Me**^ with respect to the non-methylated parent **7** (Fig. 5b). Indeed, it was possible to quantitatively obtain the neutral (non-ionic) compound by treating **7**^**Me**^**·**HPF_6_ with DBU at 70°C in acetonitrile solution. This acid-base reaction was proved to be reversible.

**Fig. 7:**
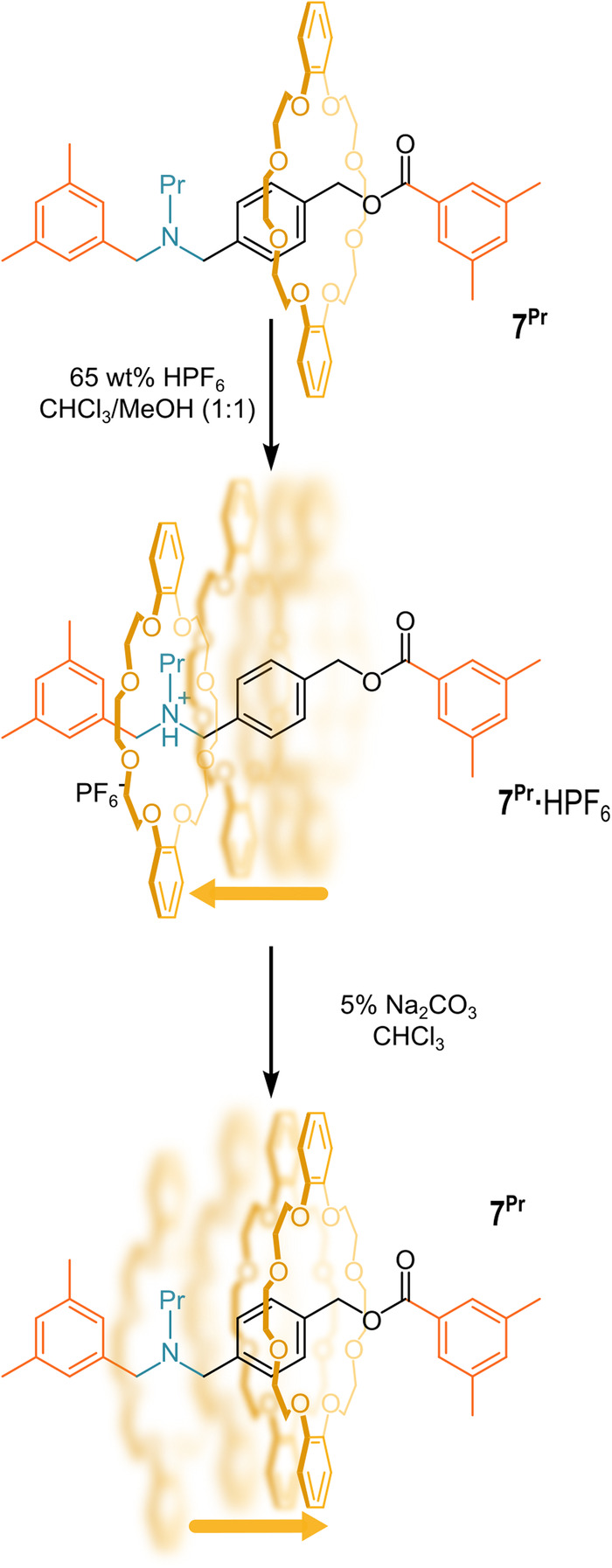
Crown ether shuttling motion. Shuttling behavior of **7**^**Pr**^**·**HPF_6_ upon acid or base treatments.

In a follow-up paper, a series of tertiary-amine [2]rotaxanes were synthesized employing a reductive *N*-alkylation on **7·**HPF_6_^73^. It was shown that **7**^**Pr**^**·**HPF_6_ (where Pr stands for propyl) allows a shuttling of the crown ether along the axle, which can be controlled by the addition of an acid or a base (see Fig. 7). In the neutral form, the wheel tends to move away from the tertiary-amine moiety. This is probably due to the repulsion between the lone pairs of the crown ether oxygens and one of the nitrogen atoms of the tertiary amine, as well as the steric hindrance of the propyl chain. The peculiarity of this tertiary ammonium/amine system is that the shuttling behavior of the wheel occurs easily despite the absence of a second cationic station on the axle. Thus, the molecule can be switched at will from the non-ionic to the ionic form through acid-base stimuli.

While the neutralization of a crown ether-based rotaxane featuring a tertiary ammonium group is easily achievable, the same does not hold true for the species bearing secondary-ammonium sites. It is worth mentioning that Stoddart and coworkers^74^, and later Leigh and Thomson^75^, reported examples of [2]rotaxanes with two secondary-ammonium stations on the axle in which only one of the ammonium sites could be deprotonated, with the other one remaining protonated and tightly bound to the crown ether. Thus, a neutral [2]rotaxane made of a crown ether wheel and a bis-benzylamine axle, remained an elusive species, and a challenge to pursue.

**Fig. 8:**
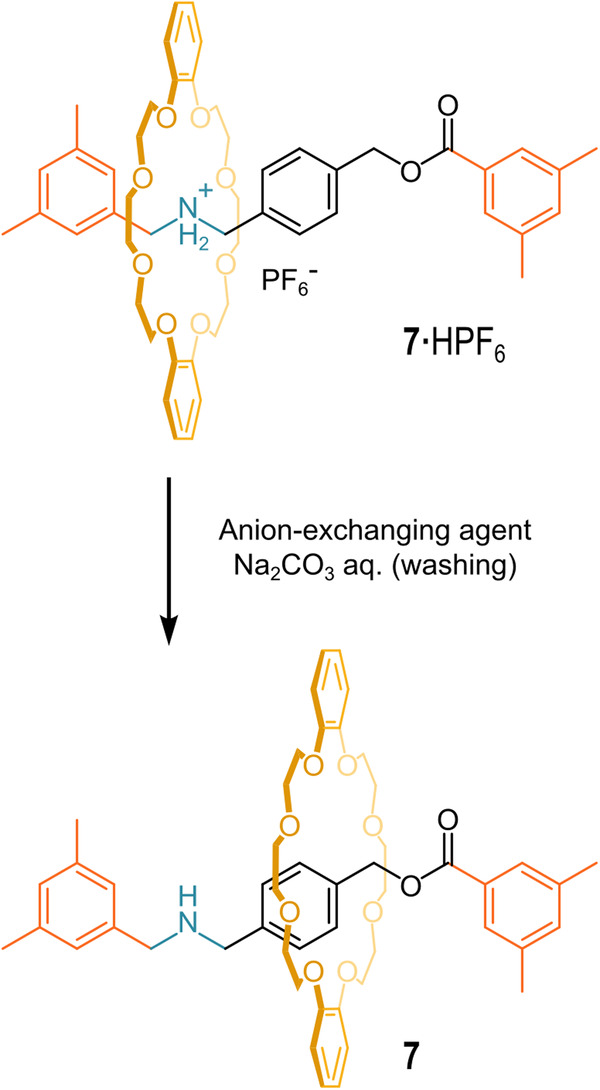
Anion exchange strategy. Anion exchange and neutralization of **7·**HPF_6_.

Ten years after the paper on the “*unusually lowered acidity*” of secondary-ammonium-based rotaxanes, Nakazono and Takata successfully neutralized rotaxane **7·**HPF_6_, using two different strategies^76^. They proposed to adopt the term “*rotaxane effect*” to identify the large thermodynamic stabilization (reflected in a very low acidity) of the secondary-ammonium moiety due to the strong interaction with the crown ether wheel. The first strategy involved an exchange with a competitive counter ion (small and hard) to weaken the strong hydrogen bond interaction between the crown ether wheel and the secondary-ammonium moiety (Fig. 8). Such a strategy was suggested by the evidence that the parent pseudo-rotaxanes do not assemble when the counteranion is a chloride ion^77,78^. Thus, the hexafluorophosphate anion (PF_6_ˉ) of **7·**HPF_6_ was quantitatively replaced with the fluoride anion (Fˉ). Eventually, the long-craved neutral [2]rotaxane **7** was isolated with a 97% yield. It is important to note that it was not possible to isolate the hydrofluoride **7** salt. The authors also successfully explored the exchange with chloride (80% conversion) and bromide anion (44% conversion).

**Fig. 9:**
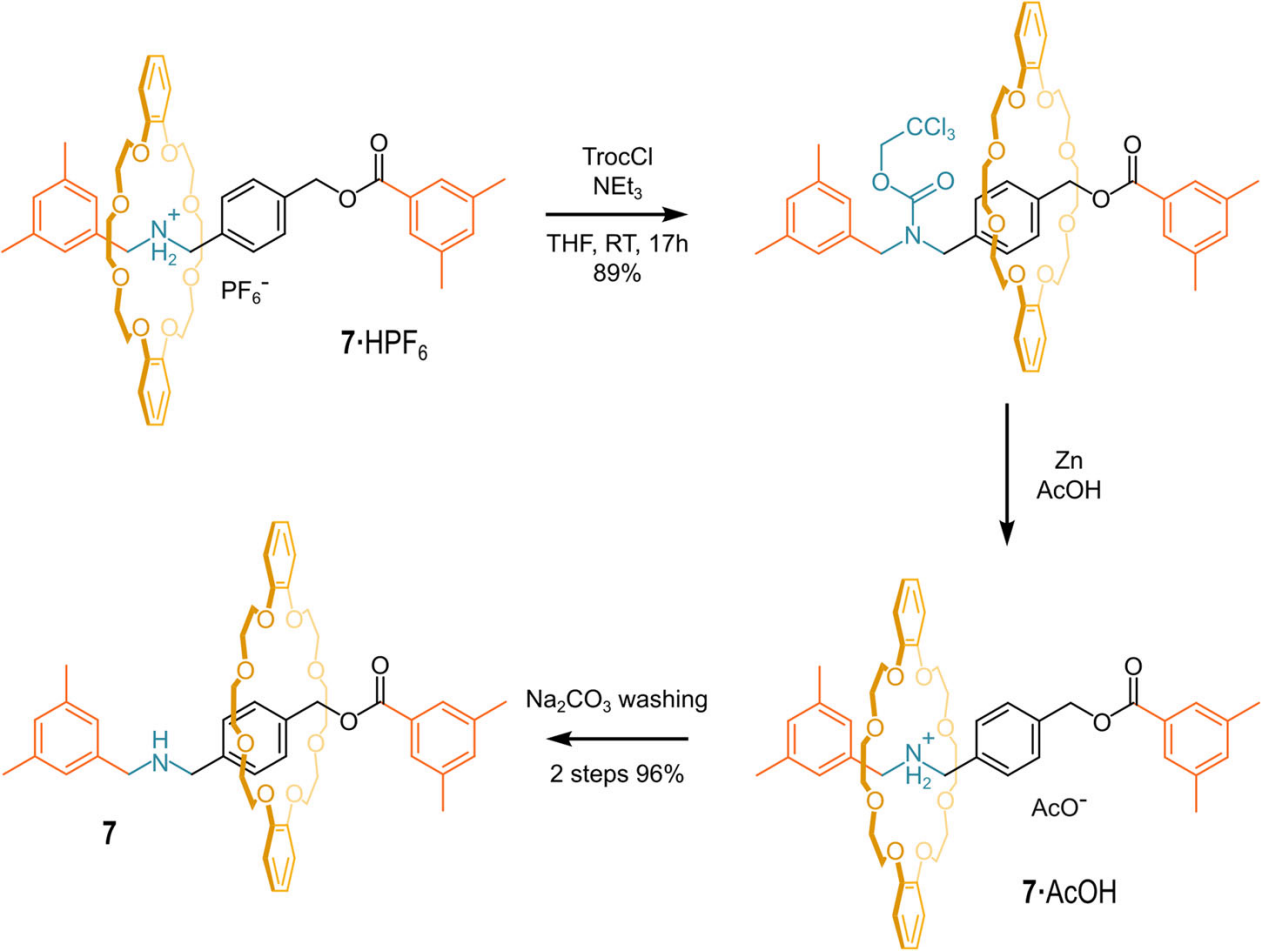
Protection strategy. Protection/deprotection sequence for the neutralization of **7·**HPF_6_.

The second strategy consisted of three main steps: (i) *N*-protection of **7·**HPF_6_ with 2,2,2-trichlorethyl chloroformate to give the corresponding carbamate, (ii) deprotection with metallic zinc in acetic acid to give **7·**HOAc, and (iii) washes with aqueous sodium carbonate, to obtain neutral **7** (see Fig. 9). This strategy relies on the above-mentioned chance to *N*-functionalize the secondary-ammonium site on the axle, and contextually have the anion exchange.

The authors additionally exploited such methods to successfully obtain a neutral [3]rotaxane.

The ^1^H-NMR study of **7** showed that the crown ether wheel is located at the central phenylene moiety on the axle, similarly to what occurs to the *N*-acetylated rotaxane. Also, variable temperature ^1^H-NMR studies showed that while at low temperatures (−50 °C) the wheel is localized on the *O*-benzyl function (of the central phenylene), at higher temperatures (+50 °C) the wheel is free to rapidly move along the axle. This temperature-dependent behavior was not observed in **7·**HPF_6_, nor in the case of the above-discussed *N*-alkylated (non-ionic) rotaxanes.

**Fig. 10:**
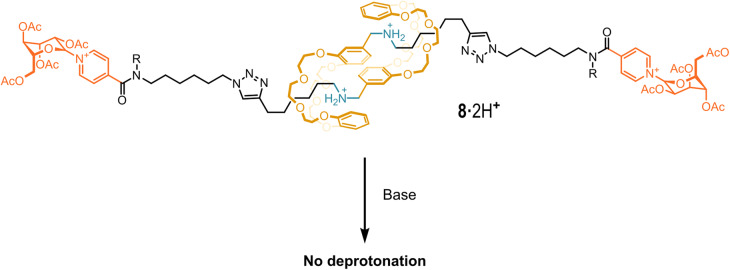
A molecular muscle. Structure of the molecular muscle **8**·2H^+^ with distal second cationic (pyridinium) stations.

In the same year (2010), Coutrot and coworkers reported an extensive study on some rotaxane-based molecular machines, defined as molecular muscles^79^. The architectures of such molecular muscles consist of rotaxanes connected to one another. One of these molecules - rotaxane **8 -** is depicted in Fig. 10. These molecules are able to contract or extend under the influence of a proper stimulus. What is of interest in our context is that it was observed that if the second cationic station (pyridinium moieties in this case), which normally facilitates the deprotonation (*vide supra*), is located too far from the secondary-ammonium station binding the crown ether wheel, the neutralization of the latter ammonium moiety is still an arduous task. Hence, the presence of an “auxiliary” second cationic station is not per se a sufficient condition for the easy deprotonation of a secondary-ammonium function. The intra-station distance must be sufficiently short as to be traversed by the crown ether wheel. Furthermore, as reported in a later work^80^, in order to assist the deprotonation, the second cationic station must be free from other already existing interactions.

At this point, it was clear that the movement of the crown ether wheel along the axle of a two-station rotaxane played a fundamental role in determining the acid-base properties of the secondary-ammonium moiety present in the axle. In this wake, in 2016, Ragazzon, Credi, and Colasson introduced a new method to quantitatively investigate the co-conformational equilibria of a [2]rotaxane consisting of a dibenzo-24-crown-8 wheel and an axle featuring two cationic stations, namely an ionic methyltriazolium and a pH-responsive secondary-ammonium one^81^. In the protonated form, the crown ether wheel was almost completely found on the secondary-ammonium site, while it resided preferentially on the methyltriazolium site after deprotonation. The authors observed that the acidity of the pH-sensitive site depended on the affinity of the wheel for the non-pH-responsive methyltriazolium station. Specifically, by performing UV-absorption-monitored acid-base titrations, they found that the acidity of the ammonium station can vary by at least 7.6 pK_a_ units upon changing the wheel position from one site to the other. This observation paved the way for the rational design of rotaxanes characterized by an on-demand pK_a_H value of the pH-responsive site, obtainable by a proper adjustment of the auxiliary cationic station.

**Fig. 11:**
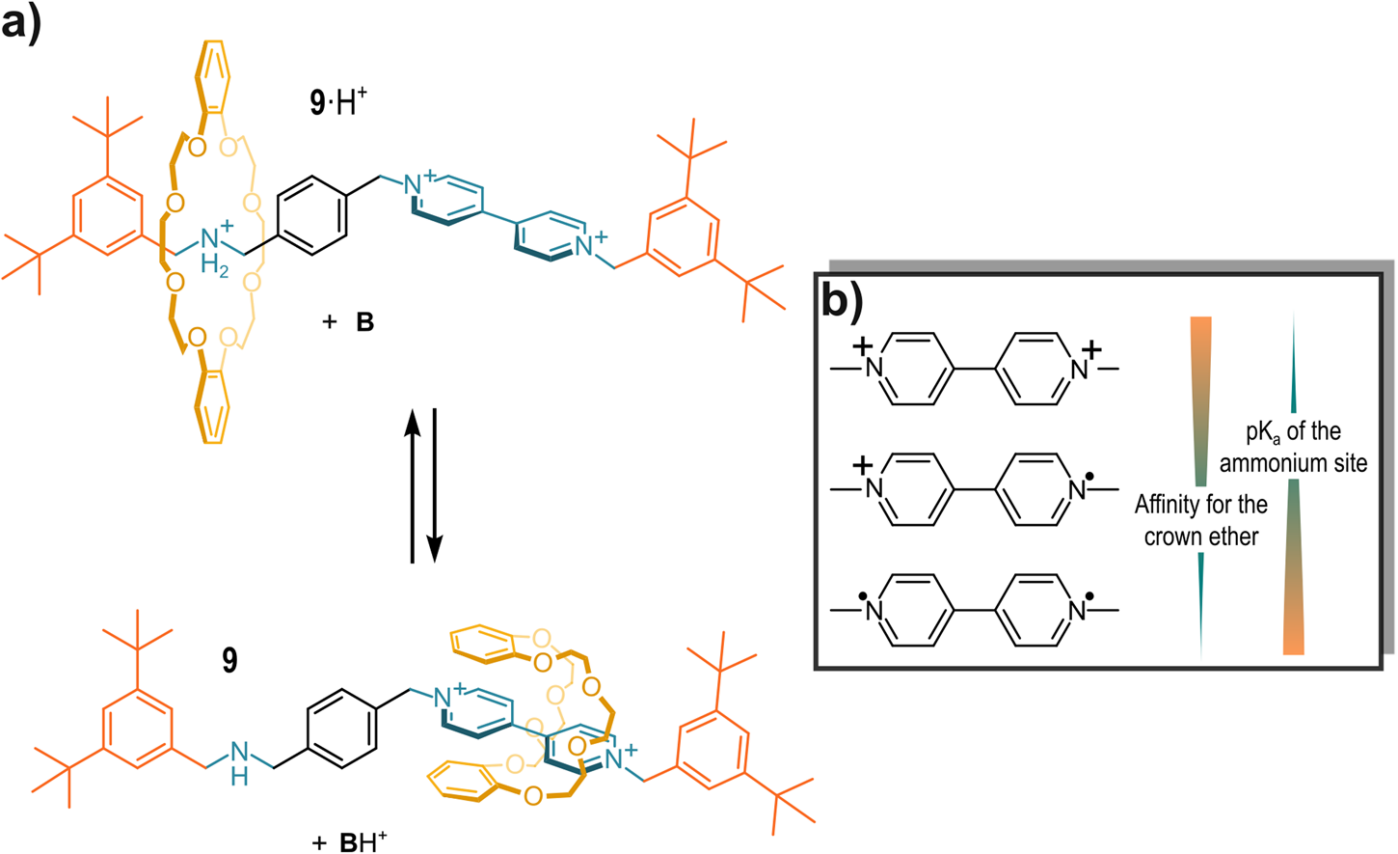
Redox-sensitive rotaxane 9. **a** Co-conformation of the [2]rotaxane **9** upon deprotonation. **b** Different charge states of the bipyridinium moiety.

Therefore, in a subsequent work, Credi, Lucarini, and coworkers exploited the [2]rotaxane **9**^67^ in which the pK_a_H of the secondary-ammonium site could be modulated by changing the affinity of the dibenzo-24-crown-8 wheel for a 4,4′-bipyridinium (bipy^2+^) station *via* electrochemical stimulation of this site (see Fig. 11a)^82^. As seen in previous examples, in the protonated form the wheel is located on the secondary-ammonium site. Conversely, after neutralization, the crown ether is found around the bipyridinium station (this phenomenon also involves a conformational change of the wheel)^83^. The affinity of the wheel for the bipyridinium station can be varied by two subsequent single electron reductions of the bipyridinium, as follows: bipy^2+^ → bipy^+·^ → bipy^0^ (see Fig. 11b). The highest affinity between the wheel and the ammonium station is observed when the latter is in the bipy^2+^ state, and it gradually decreases upon reduction. These three possible charge states of the bipyridinium site correspond to three pK_a_ values of the ammonium moiety: 18.1, 22.0, and 25.4, respectively. These pK_a_ values were extrapolated applying a thermodynamic cycle to the system.

**Fig. 12:**
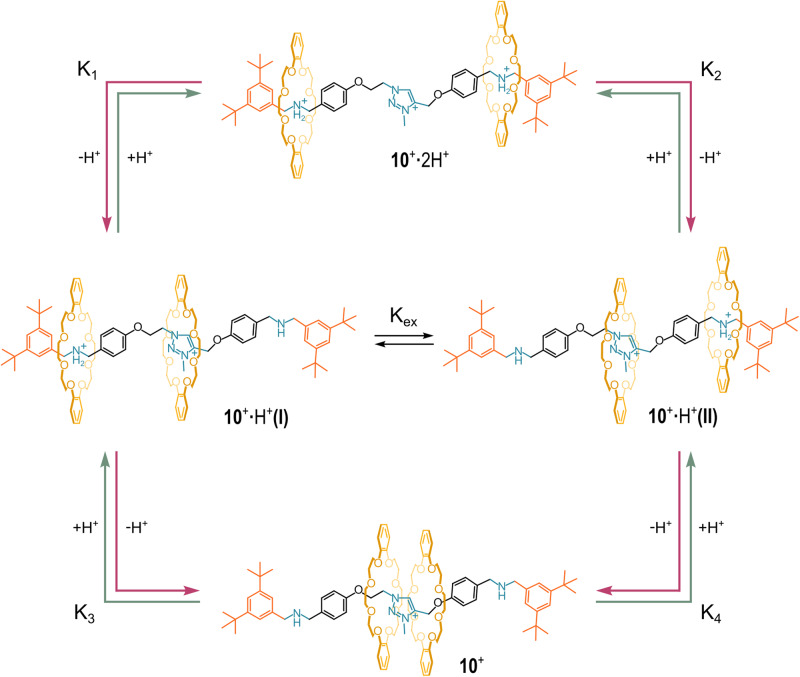
Rotaxane 10 and its acid-base equilibria. The speciation of [3]rotaxane **10**^**+**^ upon acid-base stimuli.

In 2021, Credi and coworkers published a sophisticated paper where they reported the synthesis of [3]rotaxane **10**^**+**^ consisting of two dibenzo-24-crown-8 wheels and an asymmetrical axle containing two lateral secondary-ammonium stations and a central methyltriazolium one (see Fig. 12)^84^. The number of the positively charged stations on the axle could be varied upon acid-base stimuli. Specifically, **10**^**+**^ could exhibit three, two, or one cationic stations able to bind the two crown ether wheels. In particular, the system could express four main [3]rotaxane species depending on the protonation state: (i) the tri-cation **10**^**+**^·2H^+^ in which both the two ammonium sites are protonated; (ii+iii) the two different di-cationic co-conformers **10**^**+**^·H^+^(I) and **10**^**+**^·H^+^(II), in slow equilibrium, present in a 70:30 ratio (note that the axle is asymmetric due to different spacers -CH_2_- and CH_2_CH_2_- around the imidazolium station); (iv) the mono-cation **10**^**+**^, where the central methyltriazolium moiety was the sole positively charged station on the axle. Each acid species was characterized by a specific pK_a_ value. These values were extrapolated by applying a thermodynamic cycle to the system and including the data collected from acid-base titration experiments.

**Fig. 13:**
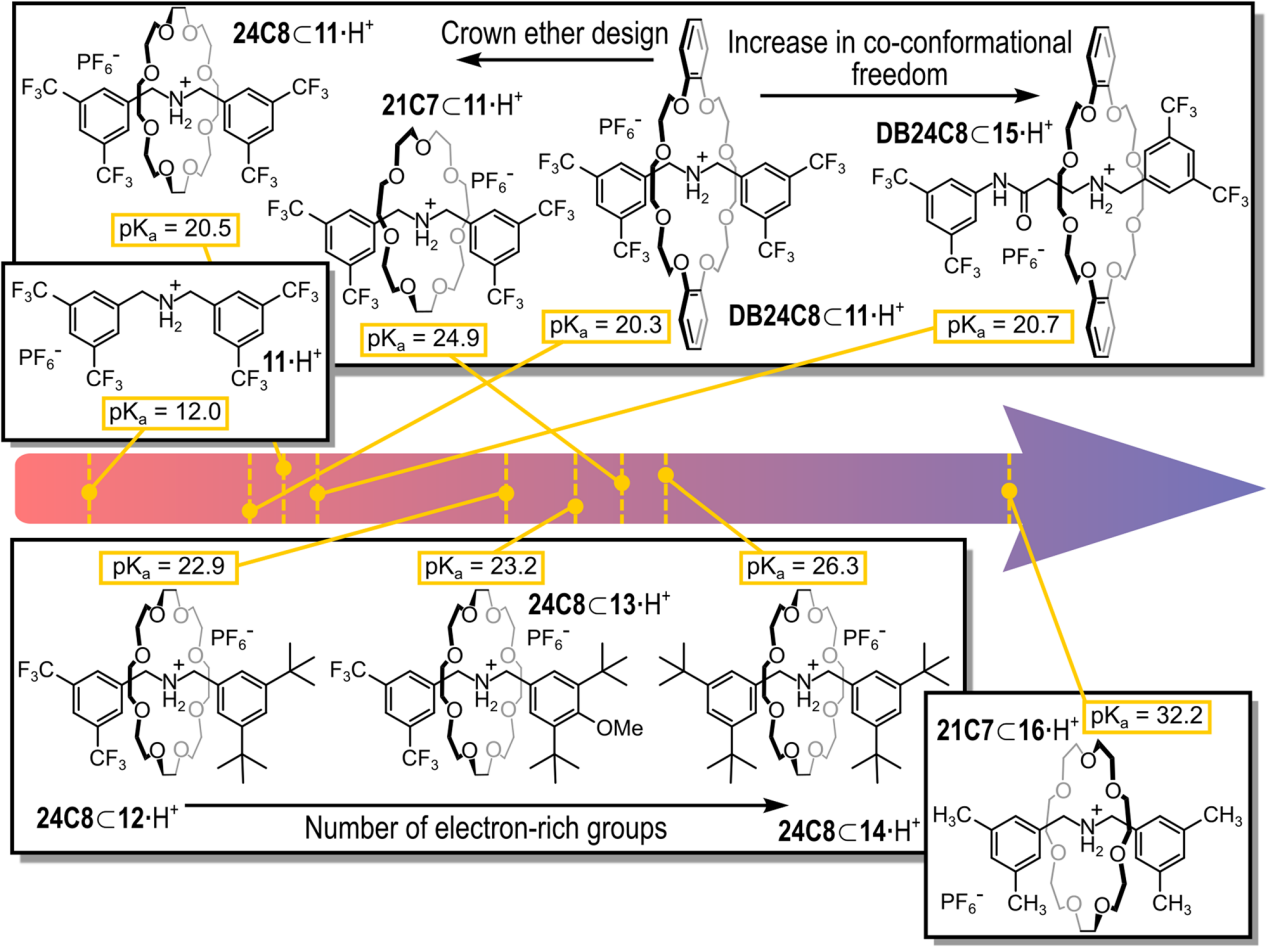
Compact rotaxane superbases. Structures and pK_a_ values of the [2]rotaxanes and the corresponding axle in the acid forms (solvent CD_3_CN, pK_a_ measured employing Lambert’s method).

The exploration of the entanglement-driven enhancement of basicity exhibited by rotaxanes found a compelling development in a work by Leigh and coworkers^85^. They exploited the strongly lowered acidity showed by secondary-ammonium [2]rotaxanes to introduce a new family of organic superbases^20^, characterized by a compact structure where the wheel is forced to stay in close proximity to the ammonium site due to the short length of the axle. The steric restriction in co-conformational freedom of the wheel provided a large basicity enhancement compared to the free axle (pK_a_H of **11** is 12.0). Figure 13 reports the structures of all the [2]rotaxanes involved in the work together with their associated pK_a_H values measured employing the Lambert method. The Lambert method allows the determination of the pK_a_ of a given species by comparison with a reference species of known pK_a_, using ^1^H-NMR spectroscopy^86^.

The rotaxanes were obtained by a metal-free active template synthesis, as depicted in Fig. 4b^87^. Taking into account the same axle, the authors observed that there were no significant differences in pK_a_H values using 24-crown-8 or dibenzo-24-crown-8 as wheels (compare **24C8⊂11** with **DB24C8⊂11**; where the ⊂ symbol indicates that axle **11** is threaded through a 24-crown-8 wheel in the first case, and a dibenzo-24-crown-8 in the second). Conversely, the pK_a_H increased dramatically when the smaller 21-crown-7 was used (**21C7⊂11**). The authors also varied the functional groups on the stoppers, taking 24-crown-8 as a standard wheel. The replacement of the trifluoromethyl groups with electron-rich *tert*-butyl groups on one stopper, largely increased the pK_a_H values (compares **24C8⊂11** with **24C8⊂12**), whereas the addition of a *p*-methoxy substituent to one of the stoppers only gave a marginal increase in pK_a_H (**24C8⊂13**). The replacement of all the trifluoromethyl groups on both stoppers with *tert*-butyl groups gave a further significant enhancement in the pK_a_H value (**24C8⊂14**). The pK_a_H of **DB24C8⊂15** was measured to be similar to that of **DB24C8⊂11**. Apparently, the effect of an elongated axle is compensated by the increased distance of one of the electron-withdrawing stoppers from the basic site. The most basic rotaxane of the series was **21C7⊂16**. Its strong basicity was attributed to the presence of the electron-rich stoppers that, due to their steric hindrance, force the lone pairs of the crown ether oxygen atoms and that of the secondary amine nitrogen atom of the axle to stay in close proximity, hugely destabilizing the rotaxane structure and dramatically increasing its basicity. Upon protonation, such repulsions are removed and likely substituted with stabilizing hydrogen bonding interactions between the protonated donating -NH_2_^+^- group and the donating -O- atoms present in the tight crown ether, that is still characterized by residual co-conformational freedom. The basicity of this rotaxane was close to that of the Verkade’s superbases^18^.

**Fig. 14:**
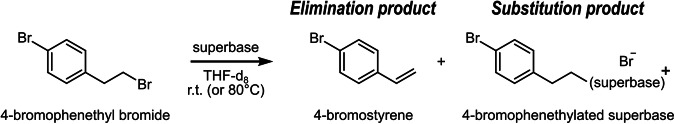
A synthetic application of rotaxane superbases. Elimination *vs* substitution reactions on 4-bromophenethyl bromide using different organic superbases.

The authors tested this new class of easily accessible superbases in a prototypical base-mediated HBr elimination reaction against other well-known superbases. Thus, 4-bromophenethyl bromide was chosen as the model substrate for evaluating the effectiveness of a given superbase in promoting the elimination over the substitution product. The results of these experiments are summarized in Fig. 14 and Table 1. The low nucleophilicity of the rotaxane superbases was evident. None of them gave the nucleophilic substitution product in appreciable amounts. This is ascribable to the large steric hindrance around the basic nitrogen atom, wrapped by the crown ether wheel. For two of the tested rotaxane superbases (**21C7⊂11** and **24C8⊂14**) the huge steric hindrance had detrimental effects on the reaction, only affording 4-bromostyrene in traces. The reaction in the presence of these two rotaxane superbases was slow even at 80 °C, suggesting that no low-energy route was accessible for a proton to reach the buried nitrogen atom of the axle. Except for these two rotaxanes, all the other tested rotaxane superbases afforded the 4-bromostyrene elimination product with excellent selectivity. Furthermore, the reactions performed using such superbases were consistently faster than those employing “traditional” superbases.

**Table 1:** Comparison between conventional and rotaxanes superbases in the elimination *vs* substitution reactions on 4-bromophenethyl bromide

Superbase	pK_a_H	Elimination/Substitution ratio	Reaction half-life at 25 °C (h)	Reaction half-life at 80 °C (h)
**24C8⊂11**	20.5	>99:1	176	
**DB24C8⊂11**	20.3	>99:1	33	
**21C7⊂11**	24.9	>99:1	>5000	4073
**24C8⊂14**	26.3	>99:1	>5000	260
**DB24C8⊂15**	20.7	>99:1	10	
**21C7⊂16**	32.2	>99:1	0.15	
DBU	24.3	87:13	635	
mTBD	25.5	>99:1	115	
P1-^*t*^Bu	27.0	>99:1	419	

*DBU* 1,8-diazabicyclo(5.4.0)undec-7-ene, *mTBD* 7-methyl-1,5,7-triazabicyclo(4.4.0)dec-5-ene, *P1-*^*t*^*Bu* *tert*-butylimino-tris(dimethylamino)-phosphorane.

The low nucleophilicity, fast deprotonation kinetics, and ease of synthesis make these rotaxane superbases attractive reagents for organic synthesis.

### Outlook

**Fig. 15:**
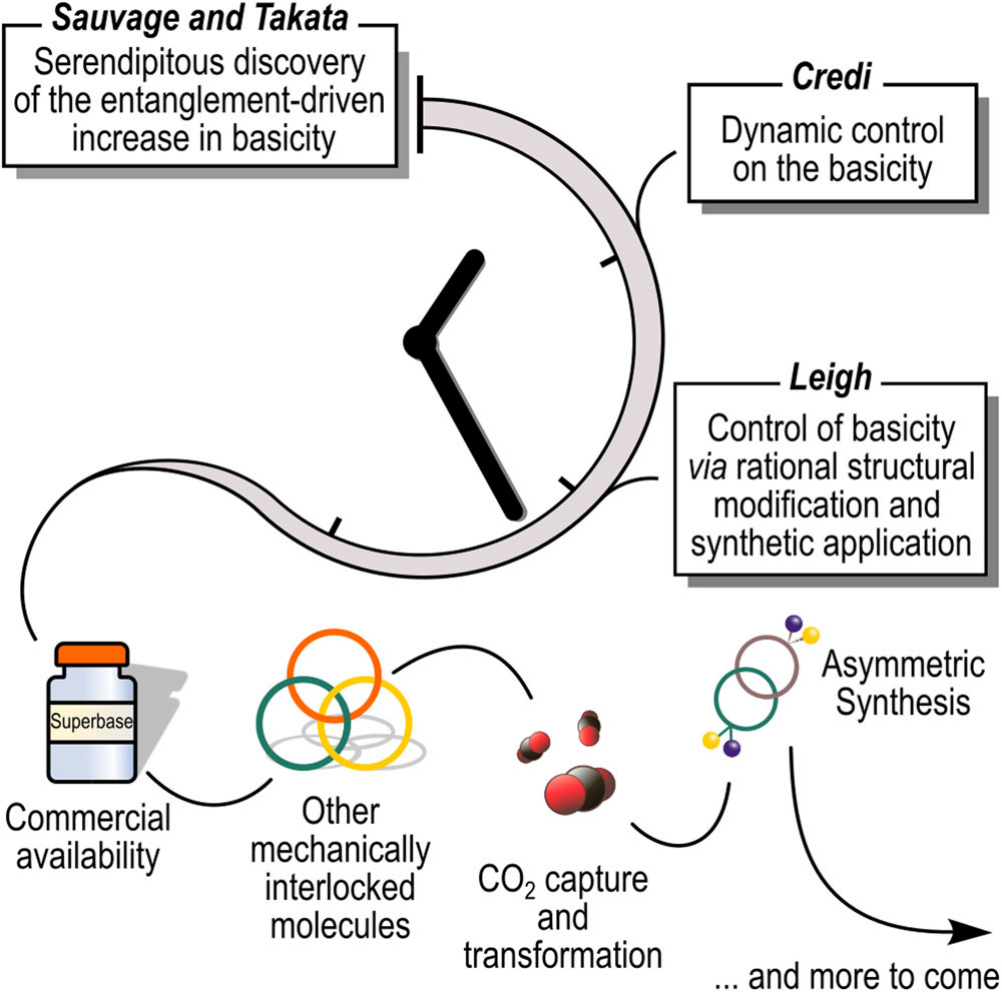
Outlook. Past and possible future evolution of the field of mechanically interlocked bases.

Although the entanglement-driven increase in basicity is still a relatively underexplored phenomenon, it is undoubtedly a fascinating one, well deserving of further study. A simple fortuitous observation has turned into a research topic thanks to the work of passionate researchers worldwide. Some of the potential implications of this effect are already evident, while others are still beyond the horizon (see Fig. 15).

The Leigh group demonstrated that rotaxane superbases can be non-nucleophilic and span a wide pK_a_H range while retaining fast protonation/deprotonation kinetics in comparison with classical superbases^85^. Since their synthesis is facilitated by the innovative *metal-free* templated approach, we believe that rotaxane superbases will soon be commercially available. This will allow their widespread use in all the fields where conventional superbases are currently employed, from organic synthesis^22–25^ to transition metal chemistry^26–28^, to CO_2_ capture^29^ and transformation^30–32^.

Furthermore, since chiral MIMs is a developing field^88,89^, new chiral mechanically interlocked superbases may be fruitfully used for many applications, including as catalysts for asymmetric synthesis.

Beyond rotaxanes (and in particular, those based on the bis-benzylammonium/crown ether motif), basicity data on other MIMs such as catenanes, molecular knots, and Borromean rings is regrettably extremely scarce.

Thus, we propose that future reports of new mechanically interconnected molecules featuring basic sites should be accompanied by an estimate - or measurement, whenever possible - of their pK_a_H. Recording basicity data for MIMs of different structures will be beneficial for the quantitative analysis of the phenomenon. It will help build a knowledge base to support the design of strongly basic entangled molecules. Additionally, any future application of these compounds will no doubt benefit from a large pK_a_H database.

Moreover, the origin of the enhancement of basicity in rotaxanes should be properly assessed. That is, an experiment should be performed to evaluate the contributions to the basicity increase from the stabilization of the protonated species and the destabilization of the unprotonated rotaxane. A comparison of pK_a_H values between a rotaxane, a pseudo-rotaxane featuring the same axle, and the non-interlocked, stoppered thread in the presence of the macrocycle should be made. If the pseudo-rotaxane displays a higher basicity than the non-interlocked system, then the stabilization of the protonated form is evident. However, the fact that pseudo-rotaxanes can spontaneously assemble in solution from a protonated thread and a wheel^77^ suggests that, in this case, the protonated pseudo-rotaxane is more stable than the protonated, unentangled thread.

## Conclusion

In the previous sections, we showed that mechanically interlocked molecules, specifically catenanes, and rotaxanes, can exhibit a much higher than expected basicity due to a molecular entanglement. In most cases, the mechanical bond among the subunits of the molecule is exploited to elicit strong electronic repulsions among lone pairs in the unprotonated state that are removed upon proton addition. In this process, the loss of strong destabilizing interactions leads to an enhancement in basicity, giving rise to a new kind of superbases (*mechanically interlocked superbases*) characterized by a through-space proximity among the basic centers, conceptually different from the through-bonds proximity which characterizes the classical superbases. We chose a historical narration which illustrates that, while in the first reports the high basicity of the MIMs was found essentially as a serendipitous feature of the molecules, in the most recent papers it appears as a rationally sought property, which opens the way to fine control of basicity (pK_a_H of the base) and synthetic applications.
